# Is the Grass Greener on the Other Side? A Qualitative Comparison Study of Psychiatry Trainee Views in England Compared to New Zealand

**DOI:** 10.1192/bjo.2022.108

**Published:** 2022-06-20

**Authors:** Neha Bansal

**Affiliations:** NHS Lothian, Edinburgh, United Kingdom

## Abstract

**Aims:**

The Royal College of Psychiatrists census (2019) highlighted that 10% of all consultant psychiatrist roles remain unfilled. This pattern is replicated elsewhere in the UK with 7.8% in Northern Ireland, 9.6% in Scotland and 12.7% in Wales. This increase in consultant vacant posts is indicative of the recruitment challenges to psychiatry. On the other hand, the 2017 New Zealand Medical Workforce survey report showed recruitment to psychiatry was up by 8.2% in 2018 compared to 2017. I conducted a qualitative comparison study to look at psychiatry trainee views regarding their training in a UK and New Zealand deanery at similar stages of their psychiatric training.

**Methods:**

Questionnaires were distributed to current psychiatry trainees in the Capital and Coast District Health Board (CCDHB) based in Wellington, New Zealand and Birmingham and Solihull Mental Health Foundation Trust (BSMHFT), UK who were between years 1–3 of their psychiatry training. Qualitative information was collated from the questionnaires regarding various aspects of their training. Areas of focus were; pros and cons of psychiatry training, suggestions for improvements, supervision, access to annual leave and study leave, teaching, encouragement to attend courses and involvement in research.

**Results:**

Of the 33 current trainees working in CCDHB, 48% were immigrants from the UK, previously having worked in the NHS.

17% of BSMHFT trainees felt valued in their organisation, compared to 64% in New Zealand.

27% in New Zealand considered switching to another training programme, whereas none considered switching in the UK. Burn out was quoted as a problem in both New Zealand and the UK. 100% were able to take annual leave with ease in New Zealand, compared to 0% in BSMHFT.

**Conclusion:**

This small study gives a closer insight into the views of trainees in New Zealand, a place often thought as being more attractive for doctors to work in. What this study shows is 2 key factors; there are shocking differences in the quality of trainee experiences between New Zealand and the UK, however New Zealand is not free from issues around trainee retention, although the study does show overall trainee satisfaction being greater in New Zealand. Feeling valued, supported and leading a life with better work-life balance appear to be key driving factors for UK graduates leaving the UK and there is more that could be done to make trainees in the UK feel more valued and prevent burn out.

